# Pharmacokinetic properties of a novel inosine analog, 4′-cyano-2′-deoxyinosine, after oral administration in rats

**DOI:** 10.1371/journal.pone.0198636

**Published:** 2018-06-06

**Authors:** Mai Hashimoto, Kazuaki Taguchi, Takako Ishiguro, Satoru Kohgo, Shuhei Imoto, Keishi Yamasaki, Hiroaki Mitsuya, Masaki Otagiri

**Affiliations:** 1 Faculty of Pharmaceutical Sciences, Sojo University, Ikeda, Kumamoto, Japan; 2 Center for Clinical Sciences, National Center for Global Health and Medicine, Toyama, Shinjuku, Tokyo, Japan; 3 DDS Research Institutes, Sojo University, Ikeda, Kumamoto, Japan; 4 Department of Infectious Diseases and Hematology, Kumamoto University, School of Medicine, Kumamoto, Japan; 5 Experimental Retrovirology Section, HIV and AIDS Malignancy Branch, National Cancer Institute, National Institutes of Health, Bethesda, Maryland, United States of America; University of Pittsburgh, UNITED STATES

## Abstract

4′-cyano-2′-deoxyinosine (SK14-061a), a novel nucleoside analog based on inosine, has antiviral activity against the human immunodeficiency virus type 1 that has the ability to acquire resistance against many types of reverse transcriptase inhibitors based on nucleosides. The aim of this study was to investigate the pharmacokinetics studies after its oral administration to rats. For this purpose, we first developed and validated an analytical method for quantitatively determining SK14-061a levels in biological samples by a UPLC system interfaced with a TOF-MS system. A rapid, simple and selective method for the quantification of SK14-061a in biological samples was established using liquid chromatography mass spectrometry (LC-MS) with solid phase extraction. The pharmacokinetic properties of SK14-061a in rats after oral administration were then evaluated using this LC-MS method. SK14-061a was found to be relatively highly bioavailable, is rapidly absorbed from the intestinal tract, and is then mainly distributed to the liver and then ultimately excreted via the urine in an unchanged form. Furthermore, the simultaneous administration of SK14-061a with the nucleoside analog, entecavir, led to a significant alteration in the pharmacokinetics of SK14-061a. These results suggest that the SK14-061a has favorable pharmacokinetic properties with a high bioavailability with the potential for use in oral pharmaceutical formulations, but drug-drug interactions should also be considered.

## Introduction

Nucleoside/nucleotide-based compounds have been approved by the US Food and Drug Administration (FDA) and are widely used in the clinics for the treatment of various types of disorders, including hepatitis B, human immunodeficiency virus type 1 (HIV-1) and cancer [1,2]. Although therapy based on these nucleoside/nucleotide-based compounds have made positive contributions to human health and welfare, the long-term treatment with these drugs frequently leads to the appearance of certain virus/cell variants that have acquired resistance against these types of compounds. These drug-resistant viruses/cells show a high sensitivity against other nucleoside/nucleotide-based compounds that have slightly different structures. In fact, 4′-CN-2′-deoxyguanosine, which was developed as a novel nucleoside analog (NA) for the treatment of the hepatitis B virus (HBV), was found to be highly active against, not only wild-type HBV, but also an HBV variant with an acquired resistance against adefovir dipivoxil (HBVA181T/N236T) and entecavir (ETV) (HBVL180M/S202G/M204V) [3]. Therefore, developing novel nucleoside/nucleotide-based compounds would be a promising strategy for overcoming the worldwide health problems associated with nucleoside/nucleotide-based drug-resistant virus/cell.

**Fig 1:**
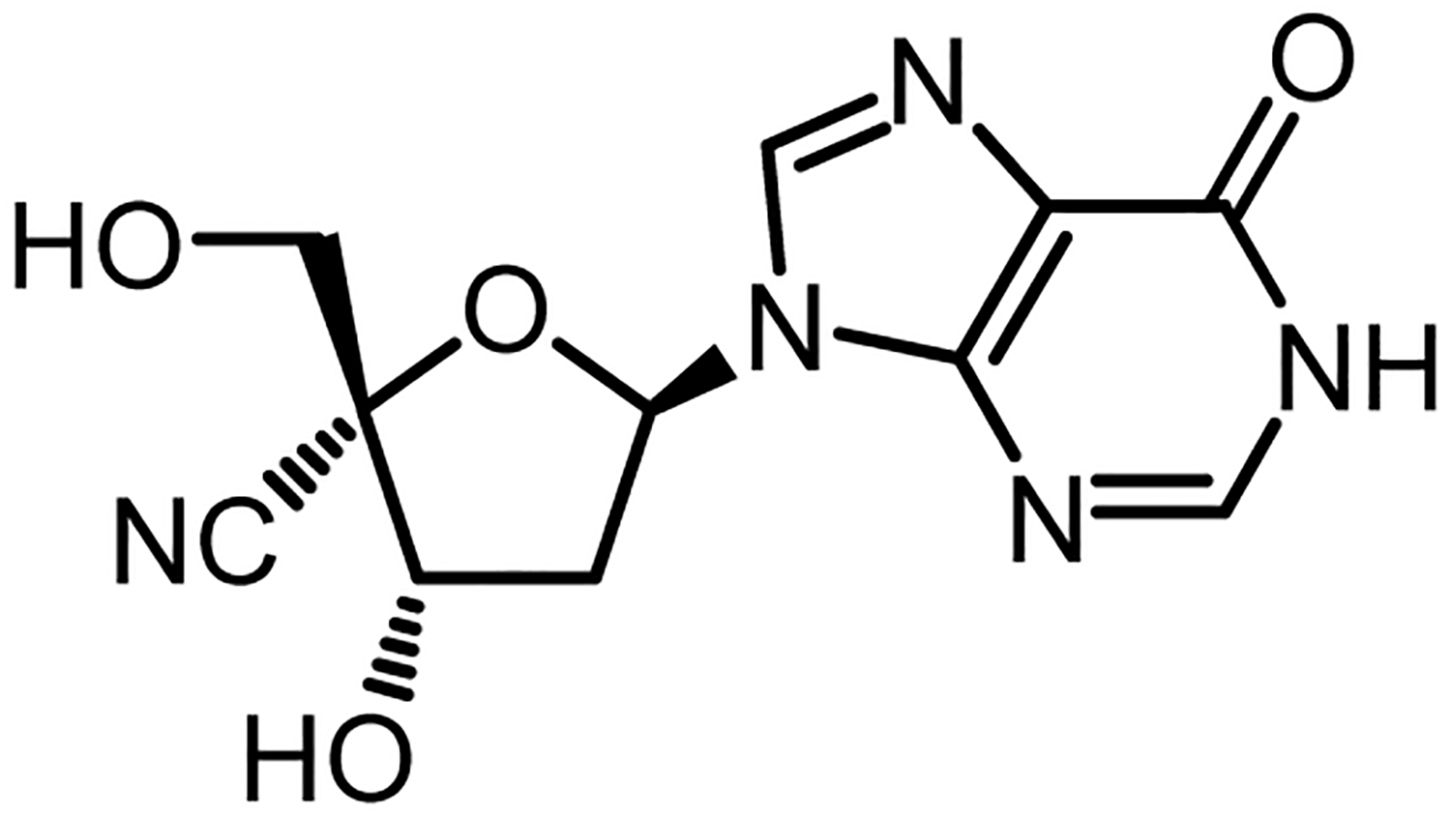
Chemical structure of 4′-CN-2′-deoxyinosine (SK14-061a).

Inosine is a naturally occurring compound with a purine structure. Along with adenosine- and guanosine-based compounds [3,4], inosine-based compounds were also synthesized and investigated for their potent anti-virus activities [4,5]. 4′-cyano-2′-deoxyinosine (SK14-061a, Fig 1) was synthesized as a nucleoside analog reverse transcriptase inhibitor (NRTIs) with potent activity against a multi drug-resistant infectious clone (HIV-1MDR), which contains five amino acid substitutions (Ala62Val, Val75Leu, Phe77Leu, Phe116Tyr and Gln115Met) and has acquired resistance against 4 commercially available NRTIs against HIV-1 (zidovudine, didanosine (ddI), zalcitabine and stavudine [6]), and HIV-1M184V, which has an acquired resistance against lamivudine [4]. At the development stage of a new drug, clarifying the pharmacokinetic properties provides us with useful information regarding pharmaceutical formulations and the dosage regimen. In fact, pharmacokinetic studies of ddI, an NRTI based on the inosine nucleoside, revealed that it has a low bioavailability due to its unstable characteristics in acidic solutions. To solve this problem, an enteric-coated capsule formulation of ddI was developed and is commercially available in clinics [7]. These facts prompted us to clarify the pharmacokinetic properties of SK14-061a.

In this study, we investigated the pharmacokinetic characteristics of SK14-061a after its oral administration in rats. For this purpose, we first developed and validated a rapid, simple and selective method for accomplishing pharmacokinetic studies by using liquid chromatography (LC) mass spectrometry (LC-MS) for the quantification of SK14-061a in plasma. In addition, since patients who are co-infected with HIV-1 and HBV typically receive NRTIs and NAs combination therapy in clinics, information on drug-drug interactions, when SK14-061a and commercially available NAs are co-administered, would also be required. In this regard, we selected ETV as a model NAs and carried out a pharmacokinetic study of the co-administration of SK14-061a and ETV to assess the effects of SK14-061a on the pharmacokinetics of ETV and vice versa.

## Materials and methods

### Chemicals and reagents

All reagents for LC-MS analysis, including ultrapure water, methanol and formic acid were LC-MS grade and were obtained from Wako Pure Chemical Industries (Osaka, Japan). ETV was purchased from TCI chemicals (Tokyo, Japan). Pooled human plasma was obtained from the Interstate Blood Bank Inc. (Memphis, TN). Human serum albumin (HSA) and human α1-acid glycoprotein (AGP) were purchased from Sigma-Aldrich (Tokyo, Japan). All other chemicals were of the highest grade commercially available. SK14-061a was synthesized following a previously described procedure [4].

### LC-MS conditions

LC-MS was conducted on a Waters ACQUITY UPLC system with a TOF-MS system (Xevo^®^ G2-S Tof, Waters). LC separations of SK14-061a were performed on a ACQUITY UPLC BEH Phenyl column (2.1 x 100 mm, 1.7 μm particle size, Waters) at 40°C. The mobile phases were an aqueous solution of 0.1% formic acid (solvent A) and 100% methanol (solvent B). The solvent gradient started at 95% A/5% B, changed to 5% A/95% B over 5 min after a 1 min hold. The gradient was returned to 95% A/5% B over 0.1 min and maintained for 4 min for the next run. The flow rate was 0.4 mL/min and the injection volume was 5 μl. The target mass for SK14-061a was 278.1.

### Validation assays

Calibration standards (15.625, 31.25, 62.5, 125, 250 and 500 ng/mL) were prepared using rat blank plasma that had been spiked with working solutions of SK14-061a. For the evaluation of intra-day and inter-day validation including precision and accuracy, quality control samples were prepared in the same manner as the calibration standards (20, 200 and 400 ng/mL). Intra-day validation was determined on the same day by analyzing five replicates of each quality control sample. Inter-day validation was determined on three different days. For the quantitation of SK14-061a using LC-MS, plasma samples were pretreated using a solid phase extraction (SPE) column, a Waters Oasis MCX 96 well plate (Waters, Milford, USA). The extraction protocol was as follows: an MCX 96 well plate was first conditioned with 100% methanol and then conditioned with 100% water. Plasma samples were loaded on the plate, further washed with 2% formic acid in water, followed by treatment with 5% methanol. The samples were eluted with ammonium/methanol/water (15/5/80). All collected samples were evaporated using a centrifugal evaporator. All of the dried samples were reconstituted in a methanol/0.1% formic acid (5/95) solution. SK14-061a concentrations were determined by LC-MS on the conditions described in the "LC-MS conditions" section.

### Measurement of clinical chemistry

Sprague-Dawley rats (male, 230–270 g) were purchased from the Kyudo Co., Ltd. (Saga, Japan). All animals were maintained under conventional housing conditions, with food and water ad libitum in a temperature-controlled room with a 12 hr dark/light cycle. Maintenance of the animals and the experimental procedures performed on them were carried out in accordance with NIH guidelines. All animal experiments were reviewed and approved by the Animal Care and Use committee of Sojo University (Permit #: 2015-P-020).

Eight rats were divided into two group: non-treatment group as a control (n = 4) and SK14-061a-treated group (p.o., 1 mg/kg, n = 4). At 9 hr after oral administration of SK14-061a, the venous blood samples were collected from inferior vena cava. The blood samples were centrifuged (3,000 g, 5 min) to obtain plasma for analysis of aspartate aminotransferase (AST), alanine aminotransferase (ALT), urea nitrogen (BUN) and creatinine. All plasma samples were stored at -80 °C prior to analysis by a commercial clinical testing laboratory (Oriental Yeast Co., Tokyo, Japan).

### Pharmacokinetic studies of SK14-061a

Rats were fasted for 12 h but with free access to water before dosing. Rats were orally (p.o.) administered a SK14-061a solution at a dose of 1 mg/kg. At stipulated times after the administration (15 min, 45 min, 90 min, 3, 4.5, 6 and 9 hr), venous blood samples were collected from tail vein using a heparinized syringe (0.25ml). The blood samples were centrifuged (3,000 rpm, 10 min) to obtain plasma. Urine samples were collected in a metabolic cage for 9 hr after the administration of the SK14-061a solution. After obtaining the last blood sample, all rats were sacrificed and kidneys and liver collected. To determine the time-dependent tissue distribution of SK14-061a, rats were sacrificed and organs (kidneys and liver) were collected at 1 and 3 hr after the oral administration of SK14-061a at a dose of 1 mg/kg. To calculate bioavailability, plasma concentrations of SK14-061a after intravenous administration (1 mg/kg) were also obtained at 3 min, 15 min, 30 min, 1, 3, 6 and 9 hr.

### Sample treatment for the quantification of SK14-061a by LC-MS

Before the LC-MS analysis, plasma samples that were collected for pharmacokinetic studies were treated in the same manner as described in the "Validation assays" section. A portion of the liver and kidney tissues (about 500 mg) were homogenized in water (1 mL). The homogenized samples were subsequently ultracentrifuged at 100,000 g for 1 hr, and the supernatant collected. Urine samples were centrifuged (3,000 rpm, 10 min) and the supernatants were used in the analyses. All samples derived from organs and urine were processed using a Waters Oasis MCX 96 well plate in the same manner as was used for the plasma samples before the LC-MS analysis.

### Pharmacokinetic study of ETV with and without the co-administration of SK14-061a

Rats were orally administered an ETV solution (1 mg/kg), with or without SK14-061a (1 mg/kg). At stipulated times after the administration (15 min, 45 min, 90 min, 3, 6 and 9 hr), venous blood samples were collected by means of a heparinized syringe (0.25ml). The blood samples were centrifuged (3,000 rpm, 10 min) to obtain plasma. The ETV was extracted from plasma samples using a Waters Oasis MCX 96 well plate in the same manner as was used for the SK14-061a extraction. The ETV concentrations were determined by LC-MS under the same conditions as was used to the determination of SK14-061a. The target mass of ETV was 278.1.

### Protein binding

Protein binding assays were performed following a previous reported procedure, with minor modifications [8]. In short, pooled human plasma, 600 μM HSA and 45 μM AGP were spiked with an aqueous solution of SK14-061a to produce a final SK14-061a concentration of 500 ng/mL, respectively. The unbound drug fraction was determined by ultrafiltration. Ultrafiltration was carried out using an Amicon^®^ Ultra-0.5 mL centrifugal filter unit with an Ultracel^®^-30 membrane (Merck Millipore Company, MA). Samples of 500 μL were centrifuged at 14,000 g for 10 min. The SK14-061a concentration were determined by LC-MS without the SPE method.

### Metabolism assay in vitro

SK14-061a (100 ng/ml) was incubated with the incubation mixtures (0.1 M potassium phosphate buffer (pH 7.4), 10 mM MgCl2, 1 mM EDTA, 1 mM NADP+, 10 mM glucose-6-phosphate, 10 units/ml glucose-6-phosphate dehydrogenase, and 0.2 mg/ml of rat liver microsomal protein). After a 5 min pre-incubation of the incubation mixture at 37°C, the metabolic reactions were initiated by the addition of SK14-061a. After 60 min, the SK14-061a was extracted from the incubation samples using the SPE method, and the SK14-061a concentration determined by LC-MS.

### Data analysis

All data are expressed as the mean ± S.D. Statistical analyses for multiple comparisons were determined by an analysis of variance (two-way ANOVA) using the Bonferroni correction for multiple comparisons. A probability value of p < 0.05 was considered to be significant. A non-compartment model was used for the pharmacokinetic analysis. Parameters were calculated using the program of a moment analysis developed by Tabata et al [9]. Half-life (t_1/2_) were calculated from the elimination phase. Area under the concentration-time curve (AUC) were calculated using the following equations:

$$\text{AUC}=\int_{0}^{\infty }\text{Cdt}$$

## Results and discussion

### Extraction of SK14-061a from biological samples

**Fig 2:**
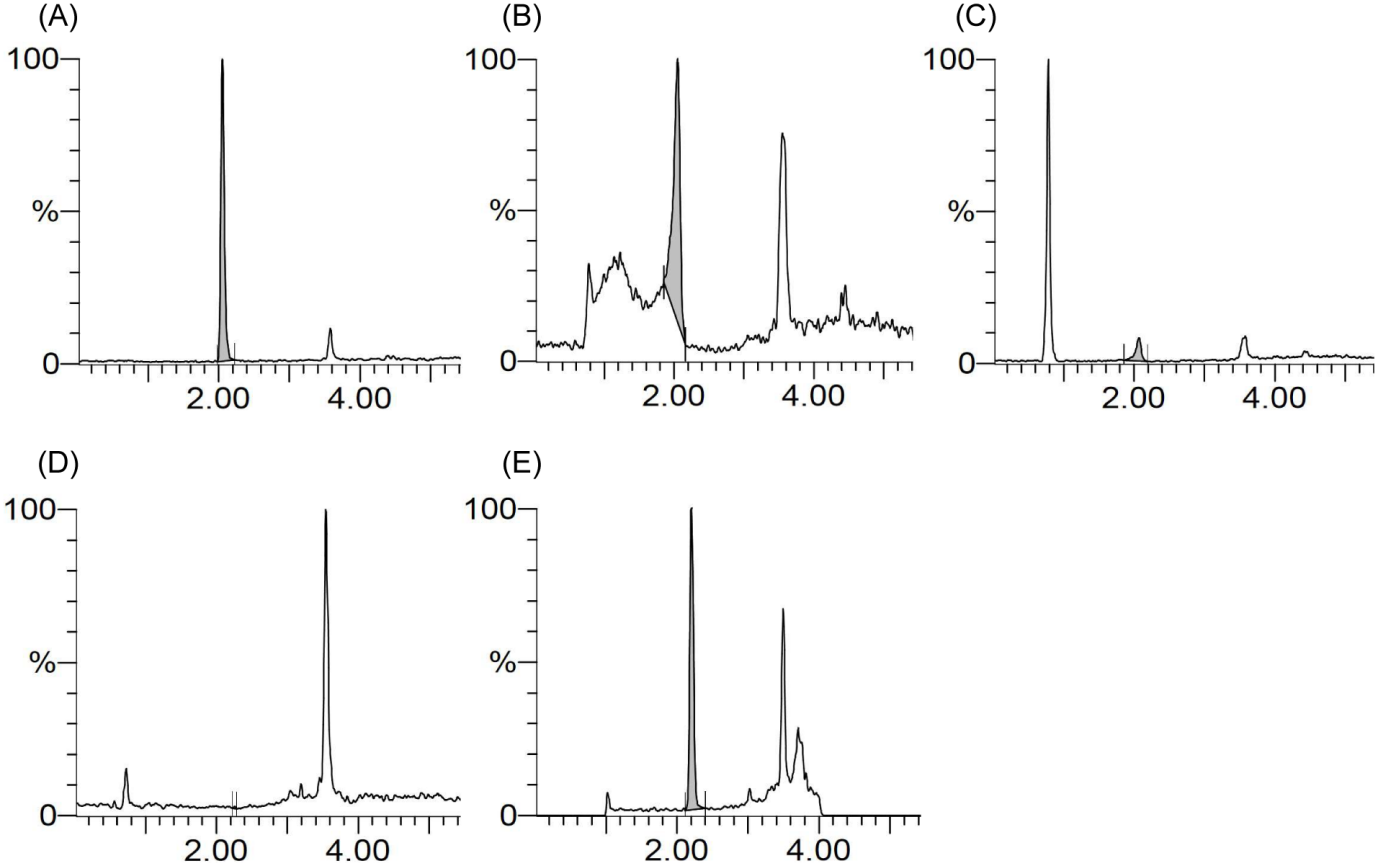
LC-MS chromatograms of (A) SK14-061a spiked with water, (B) SK14-061a spiked with plasma after a methanol treatment, (C) SK14-061a spiked with plasma after an acetonitrile treatment, (D) blank plasma after SPE treatment, and (E) SK14-061a spiked with plasma after SPE treatment. All SK14-061a samples contained 500 ng/mL in concentration before either reverse liquid-liquid extraction or SPE. The SK14-061a were detected by the LC-MS as described in the Materials and Methods section.

For the quantification of SK14-061a by LC-MS, we used a simple mobile phase consisting of formic acid/methanol (0.1/99.9) and gradient elution. Using this procedure, SK14-061a appears as a single sharp peak at around 2 min (m/z 278.1) with better ionization efficiency (Fig 2A). In addition, the measurement time for one sample was 9 min. Prior to the in vivo pharmacokinetic studies of SK14-061a, a method for extracting it from biological samples was needed. It is generally known that a reverse phase liquid-liquid extraction method is the simplest and fastest form of extraction for low molecular weight compounds. However, a reverse phase liquid-liquid extraction method using methanol and acetonitrile cause an obvious peak broadening (Fig 2B) and the disappearance of the peak (Fig 2C), respectively, indicating such a reverse phase liquid-liquid extraction method is not suitable for the extraction of SK14-061a.

In a previous study, ddI, a commercially available inosine-based NRTI, was reported to be adequately extracted from biological samples using an SPE method prior to an LC-MS analysis [10]. Thus, we examined the use of an SPE method using Waters Oasis MCX columns, which is a SPE column for extracting weakly basic compounds with cation-exchangeable groups. This SPE procedure was capable of removing most of the endogenous compounds from plasma samples (Fig 2D), and allowed the extraction of SK14-061a from plasma with a sharp peak and without any endogenous, interfering peaks (Fig 2E). In addition, the absolute recovery results for SK14-061a in plasma were 92.4 ± 10.9%, indicating that this SPE method has an acceptable extraction recovery. These results indicate that our established LC-MS conditions and the extraction method are adequate for the quantification of SK14-061a in biological samples.

### Validation assay

To apply our established method to in vivo pharmacokinetic studies of SK14-061a, a validation assay is also necessary. Thus, we next collected intra-day and inter-day validation data. A calibration curve, which was produced by plotting the peak intensity of SK14-061a (y) versus the respective standard concentration (x), showed good linearity over the concentration range 15.625–500 ng/mL by assaying calibration samples at 6 different concentrations (r^2^ = 0.99). In addition, the lower limit of quantification for SK14-061a was 15.625 ng/mL in this established method. The intra-day and inter-day precision and accuracy are summarized in Table 1. The intra-day precision ranged from 1.4% to 2.4% and the intra-day accuracy was 87.7–91.6%. The inter-day precision and accuracy range was 6.5–11.7% and 93.4–105.8%, respectively. According to the Guidelines on bioanalytical method validation, the precision should not exceed 15% and the accuracy range should be between 85 and 115% [11]. Therefore, our established LC-MS method with SPE has an acceptable level of precision and accuracy.

**Table 1:** Intra- and inter-day accuracy and precision for SK14-061a in plasma.

	Nominal concentration (ng/mL)	Measured concentration (ng/mL)	RSD (%)	Accuracy (%)
Intra-day	20	17.6 ± 0.4	2.4	87.9
	200	183.2 ± 4.1	2.2	91.6
	400	350.7 ± 4.8	1.4	87.7
Inter-day	20	19.6 ± 1.4	7.2	98.2
	200	211.7 ± 24.7	11.7	105.8
	400	373.4 ± 24.4	6.5	93.4

Each value represents the mean ± S.D. (n = 3–5)

### Pharmacokinetic studies of SK14-061a in rats

The simple, precise and accurate LC-MS method coupled with the SPE method provided the following basic pharmacokinetic information regarding SK14-061a, especially its absorption, distribution, metabolism and excretion, in rats.

Since the liver and kidney injuries influence on the pharmacokinetic properties of compounds, we investigated the toxicity of SK14-061a against the liver and kidney before pharmacokinetic studies of SK14-061a. Compared to control rats, no significant changes of aspartate and alanine aminotransferase values, which reflect liver function, and creatinine and blood urea nitrogen, which reflects renal function, were observed in rats that had been orally administered with SK14-061a at a dose 1mg/kg (S1 Fig).

**Fig 3:**
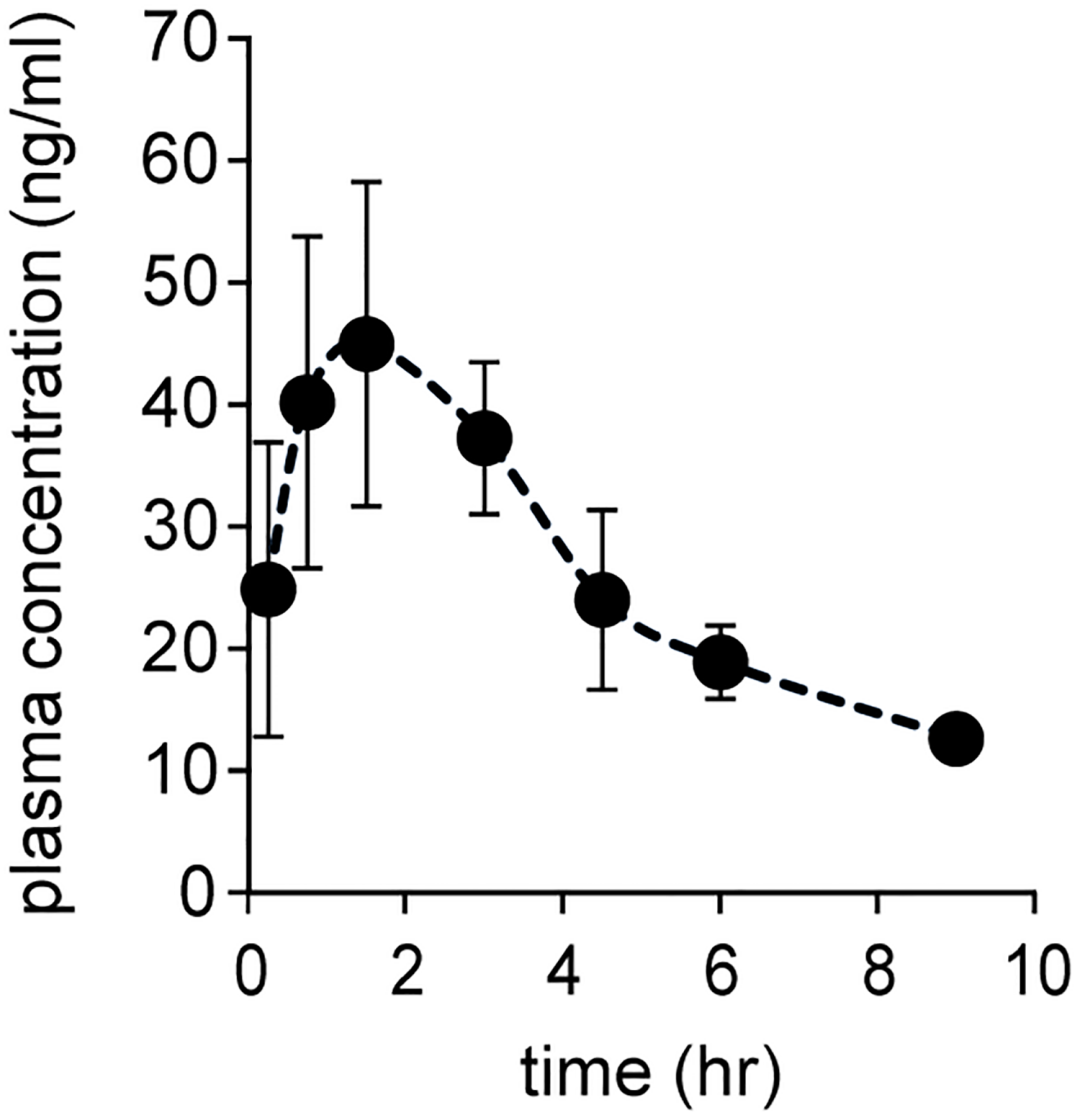
Time course for the plasma concentration of SK14-061a after oral administration at a dose of 1 mg/kg in rats. Venous blood samples were collected at 15 min, 45 min, 90 min, 3, 4.5, 6 and 9 hr after oral administration. The SK14-061a concentrations in plasma were measured by LC-MS combined with the SPE method. The values are the mean ± SD. (n = 4).

The plasma concentration time curves profiles for SK14-061a after oral administration at a dose of 1 mg/kg are shown in Fig 3. The pharmacokinetic parameters calculated from the plasma concentration curve using a non-compartmental analysis are summarized in Table 2. The half-life (t_1/2_) of SK14-061a was about 3.6 hr, with a time to reach peak plasma concentration (t_max_) value of 1.3 hr. In a previous study, ddI was reported to show site dependent absorption, with a better absorption in the duodenum than in the ileum and colon [12]. In addition, it was reported that other purine structure-based NAs, ETV, also showed region-specific apparent absorption in the rank order duodenum > jejunum > ileum, with a rapid t_max_ value [13]. It can therefore be concluded that SK14-061a is also likely to be rapidly absorbed in the duodenum as well as ddI and ETV. In addition, the plasma concentration time curves profiles for SK14-061a after intravenous injection at a dose of 1 mg/kg were also evaluated in order to calculate the bioavailability of SK14-061a (S2 Fig and Table 2). SK14-061a showed a much higher bioavailability (approximately 65%) than ddI (8–16% in rats [14,15]), indicating that SK14-061a is absorbed relatively efficiently from the intestinal tract. Also, the higher value of mean residence time (MRT) in the case of p.o. than i.v. would be attribute to the prolongation of t_1/2_ p.o. These findings suggest that SK14-061a can probably be used in the form of an oral preparation.

**Table 2:** Pharmacokinetic parameters of SK14-061a after intravenous and oral administration of a dose of 1 mg/kg in rats.

	i.v.	p.o.
t_1/2_ (hr)	3.01 ± 1.48	3.59 ± 1.20
MRT (hr)	3.58 ± 1.86	5.47 ± 1.52
AUC (hr·ng/mL)	475.4 ± 82.2	307.5 ± 49.7
CL (L/hr/kg)	2.08 ± 0.34	-
CL/F (L/hr/kg)	-	3.32 ± 1.52
t_max_ (hr)	-	1.29 ± 0.34
C_max_ (ng/mL)	431 ± 36.3	48.8 ± 11.8
F (%)	64.7	
MAT (hr)	1.89	

t_1/2_: half-life, MRT: mean residence time, AUC: area under the concentration-time curve, CL: clearance, F: bioavailability MAT: mean absorption time. Each value represents the mean ± S.D. (n = 4)

**Fig 4:**
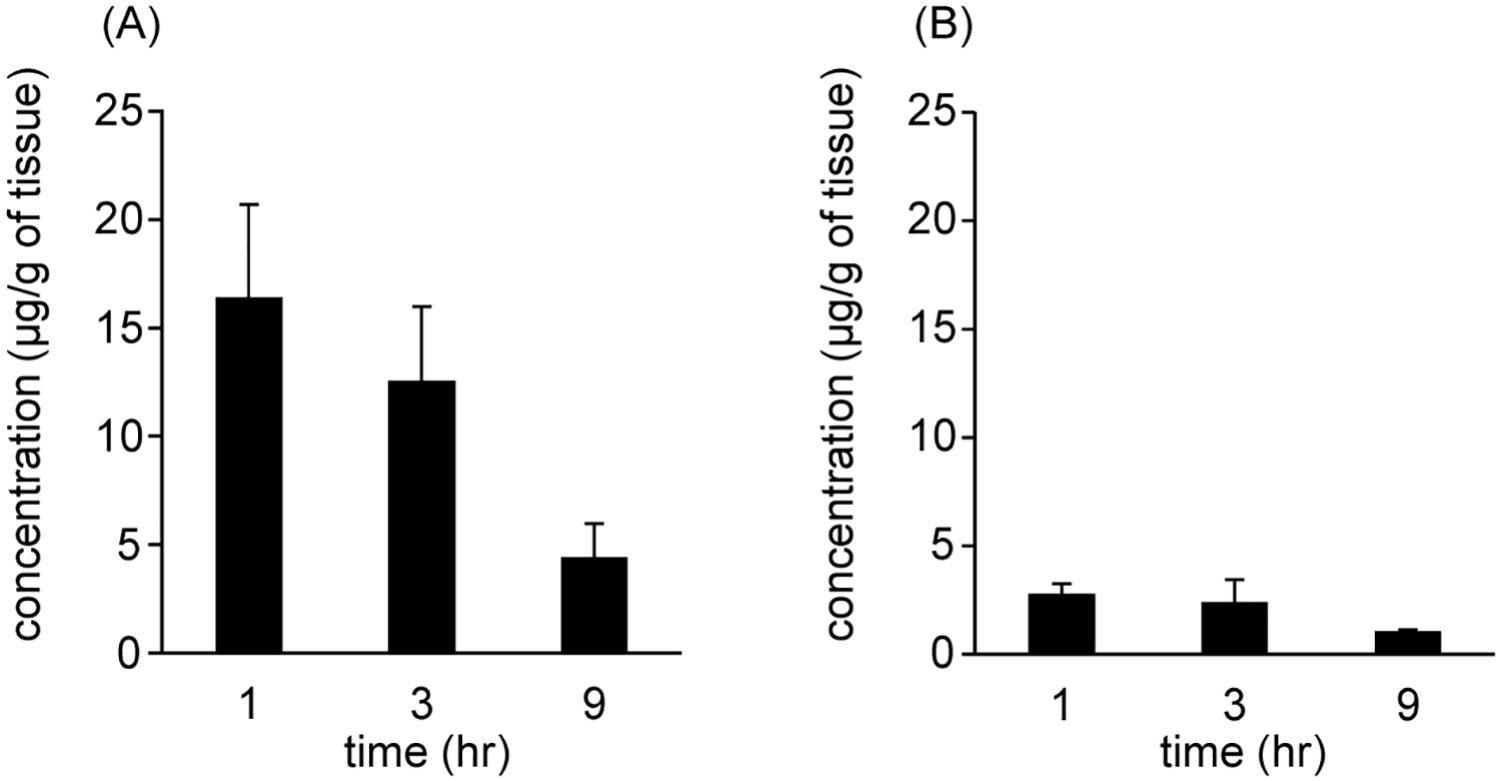
The tissue distribution to (A) liver and (B) kidney of SK14-061a at 1, 3 and 9 hr after oral administration at a dose of 1 mg/kg in rats. The values are the mean ± SD. (n = 4).

The distribution of SK14-061a in the liver and kidney were evaluated after the oral administration of SK14-061a. As shown in Fig 4, SK14-061a showed a maximum distribution in the liver and kidney at 1 hr after administration, and the concentration of SK14-061a in both organs gradually decreased until 9 hr after its administration. In addition, SK14-061a was distributed at higher levels to the liver than the kidney.

In terms of distribution, protein binding properties are also important information on pharmacokinetics because they are sometimes related to drug-drug interactions. To address this issue, we examined the protein binding properties of SK14-061a. The protein binding rate for SK14-061a in human pooled plasma was about 63.7 ± 0.04%. It is well-known that various substances, including drugs undergo noncovalent binding to albumin and AGP, when they are transported in the body [16]. Thus, we subsequently evaluated the binding properties of SK14-061a to HSA and AGP. The binding rate of SK14-061a to HSA was about 14.7 ± 0.03% and that for AGP about 22.1 ± 0.01%, indicating that SK14-061a does not bind appreciably to either HSA or AGP.

The liver produces a wide variety of enzymes such as cytochrome P450 (CYP450). Since SK14-061a is distributed to the liver (Fig 4), the possibility that SK14-061a is metabolized in the liver cannot be excluded. Therefore, we examined the issue of whether SK14-061a is metabolized by CYP, which has a broad range of functions, including the metabolism and detoxification of endogenous and exogenous substances [17]. The findings indicated that the non-metabolized form of SK14-061a was detected after incubation with rat microsomes (73.7 ± 10.7%), indicating that SK14-061a is not extensively metabolized by the action of CYP450. It is known that nucleoside/nucleotide-based compounds are not substrates for the CYP450 enzyme system [18], and no oxidative or acetylated metabolite was observed during the in vivo process [19]. In addition, inosine is endogenously metabolized to allantoin in rodents and to uric acid in humans [20]. Since SK14-061a possesses an inosine structure, it may be metabolized to allantoin-like metabolites. In fact, ddI is metabolized to uric acid or enters the purine metabolic pool [21]. Collectively, these facts may suggest that the metabolic pathway for SK14-061a is likely similar to that for other nucleoside/nucleotide-based compounds or for endogenous inosine.

**Fig 5:**
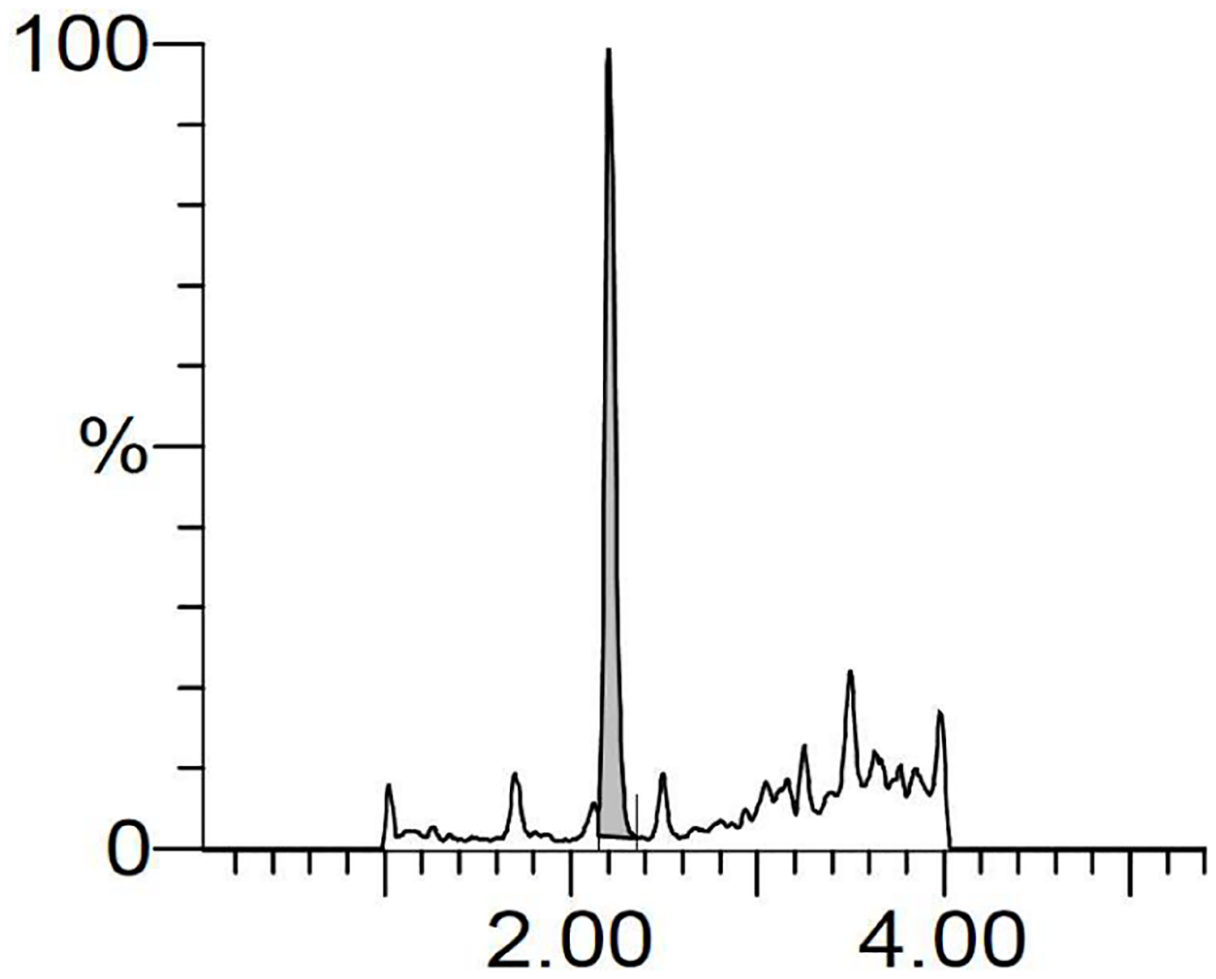
LC-MS chromatograms of SK14-061a in urine. Urine samples were collected in a metabolic cage and were pretreated on an SPE column (Waters Oasis MCX 96 well plate) for extracting SK14-061a as described in the Materials and Methods section.

We also evaluated the excretion of SK14-061a in the urine. As shown in Fig 5, the LC-MS chromatographic profile shows that a single sharp peak corresponding to SK14-061a at around 2 min (m/z 278.1) in urine sample after the SPE procedure, indicating that SK14-061a is excreted unchanged into the urine. The amount of unchanged SK14-061a that accumulated in the urine was about 46 ± 17 μg at 9 hr after oral administration, corresponding to about 20% of the administered dose. Taken together the bioavailability of SK14-061a (approximately 65%), the recovery of SK14-061a at 9 hr after oral administration was approximately 30% of the administered dose. As described in "Metabolism" section, there was a possibility that SK14-061a might have been metabolized to allantoin-like metabolites, similar to endogenous inosine. If this was the case, SK14-061a would have been excreted into urine in form of a metabolite. In addition, it is known that NAs are rapidly phosphorylated in cells, and the resulting phosphorylated derivatives are dephosphorylated relatively slowly and returned to the systemic circulation in an unchanged form [22,23]. It is therefore possible that SK14-061a would also be phosphorylated in cells after distribution to the liver, and then gradually dephosphorylated prior to its elimination via the urine in an unchanged form. In fact, in another study, we confirmed that the amount of SK14-061a gradually accumulated in the urine during 72 hr after oral administration (about 45% of the administered).

### Pharmacokinetic studies of SK14-061a and ETV co-administration

**Fig 6:**
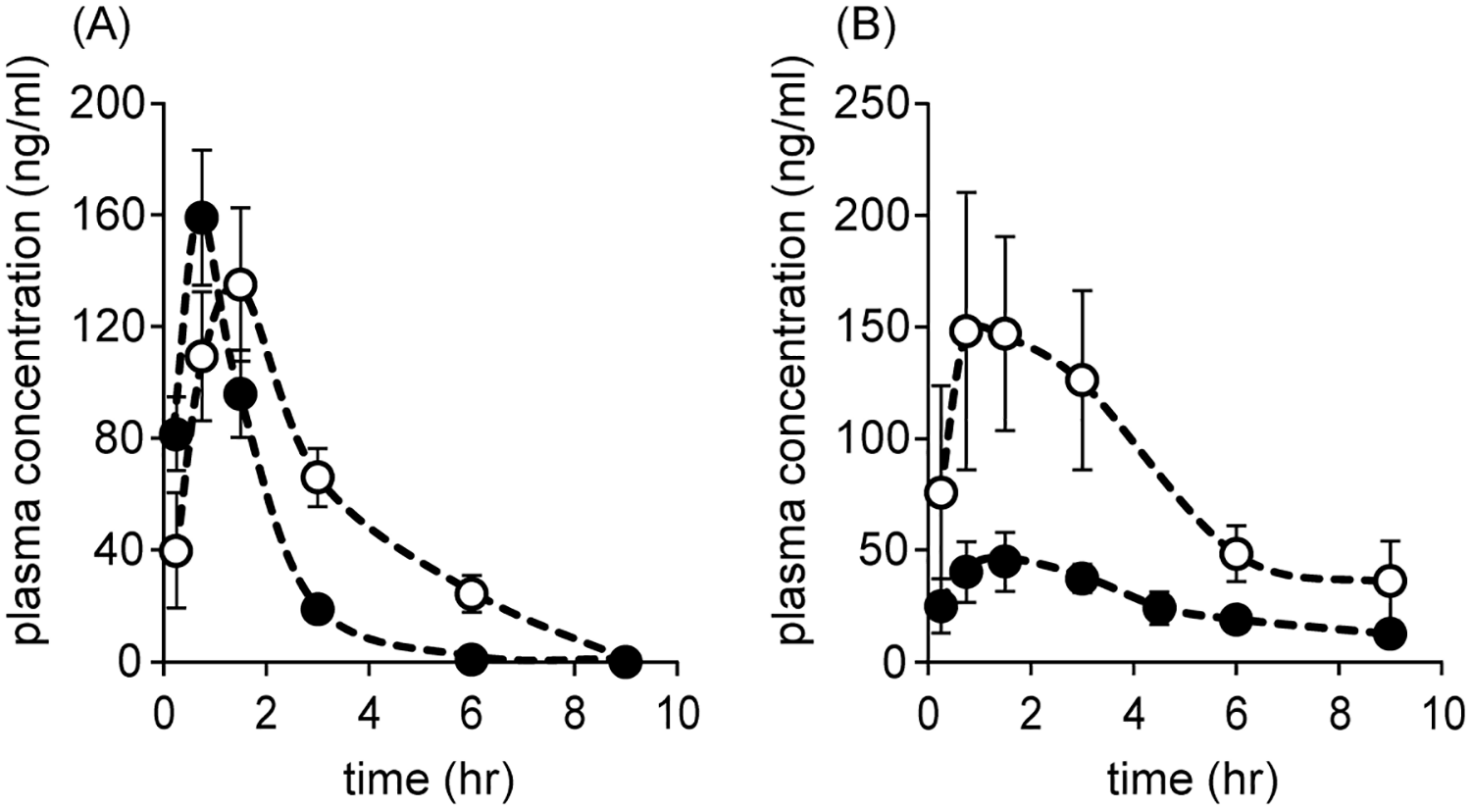
(A) Time course for the plasma concentration of ETV after an oral administration of ETV alone (closed circle) or combination with SK14-061a (open circle) at doses of 1 mg/kg in rats. (B) Time course for the concentration of SK14-061a in plasma after the oral administration of SK14-061a alone (closed circle) or combination with ETV (open circle) at doses of 1 mg/kg in rats. Venous blood samples were collected at 15 min, 45 min, 90 min, 3, 6 and 9 hr after administration. The SK14-061a and ETV concentrations in plasma were measured using LC-MS with the SPE method. The data for SK14-061a alone are quoted from Fig 3. The values are the mean ± SD. (n = 4).

Co-infection of HBV is frequently seen in 7–10% of patients with HIV in the United States and Europe and in 20–30% of patients with HIV in the Asia and Africa [24]. These patients may be treated by NRTI with NAs that have dual HBV and HIV anti-viral effects [25]. ETV has dual HBV and HIV anti-viral effects, and its use in patients with HIV and co-infected with HBV has been associated with an M184V mutation that confers resistance to lamivudine and emtricitabine [26,27]. Since SK14-061a was found to be highly active against HIV-1M184V [4], there is a possibility that ETV could be used with SK14-061a in HIV/HBV-coinfected patients. It has previously been reported that tenofovir, which has purine structure, causes the pharmacokinetic alteration of ddI when they are simultaneously administered [28,29]. These facts led us to the hypothesis that the co-administration of ETV and SK14-061a may interfere with the pharmacokinetic properties of each other. Especially, SK14-061a and ETV both have a similar purine structure. Thus, we also evaluated the effect of the co-administration of SK14-061a and ETV on the pharmacokinetic characteristics of ETV and vice versa. Fig 6A shows the plasma concentration curve for ETV after an oral administration of ETV with or without the co-administration of SK14-061a at a dose of 1 mg/kg. The t_1/2_ and under the plasma concentration-time curve (AUC) were significantly increased when SK14-061a is co-administered (Table 3). Interestingly, the t_max_ of ETV was delayed when administered in combination with SK14-061a (Fig 6A, Table 3). In the case of SK14-061a, the concentration of SK14-061a in the plasma, when administered in combination with ETV, was dramatically increased compared to the administration of ETV alone (Fig 6B). Concerning changes in the plasma concentration, the t_1/2_, C_max_ and AUC for SK14-061a in combination with ETV were increased slightly in comparison with SK14-061a being administered alone (Table 3). The t_max_ for SK14-061a was also delayed in combination with ETV, as well as in the case of ETV. The reason for this strong pharmacokinetic interaction between SK14-061a and ETV is unclear, but it could result from similarities in chemical structure (purine structure) between SK14-061a and ETV are involved in this effect. As described above, both SK14-061a and ETV are thought to be absorbed in the upper small intestine [30], suggesting that ETV may possibly promote the absorption of SK14-061a via a transporter or permeation through the intestinal membrane. In addition, multiple drug transporters are involved in the renal secretion and reabsorption of NAs [31,32], indicating another possibility that competition in the excretion pathway in the kidney might also be a factor. However, further studies will be needed to determine whether these factors are related to the pharmacokinetic interactions between SK14-061a and ETV.

**Table 3:** Pharmacokinetic parameters of SK14-061a and ETV after the oral co-administration of SK14-061a and ETV at a dose of 1 mg/kg in rats.

	SK14-061a		ETV	
	ETV (-)	ETV (+)	SK14-061a (-)	SK14-061a (+)
t_1/2_ (hr)	3.59 ± 1.20	4.07 ± 2.20	0.72 ± 0.05	1.98 ± 0.51**
MRT (hr)	5.47 ± 1.52	3.14 ± 2.16	1.35 ± 0.03	3.24 ± 0.68**
AUC (hr·ng/mL)	308 ± 49.7	972 ± 185**	261± 16.2	492 ± 31.5**
CL/F (L/hr/kg)	3.32 ± 1.52	1.06 ± 0.20**	3.85 ± 0.25	2.04 ± 0.13**
t_max_ (hr)	1.29 ± 0.34	1.07 ± 0.40	0.75	1.25 ± 0.43*
C_max_ (ng/mL)	48.8 ± 11.8	165 ± 46.6**	147 ± 5.82	135 ± 27.4

t_1/2_: half-life, MRT: mean residence time, AUC: area under the concentration-time curve, CL: clearance, F: bioavailability.

*p<0.05,

**p<0.01, Each value represents the mean ± S.D. (n = 4) The data for the SK14-061a (ETV (-)) are quoted from Table 2.

## Conclusions

The present study reports on the first hard data regarding the pharmacokinetic properties of a novel anti-HIV agent, SK14-061a, after its oral administration in rats. The results obtained from in vivo pharmacokinetic studies of SK14-061a demonstrate that SK14-061a is suitable for oral administration due to its relatively better absorption from intestinal tract, combined with sufficient plasma retention. However, SK14-061a may induce significant pharmacokinetic interactions with other drugs that has the similar structure (purine structure), when they are orally co-administered. The data obtained in this study provide basic information regarding the development of inosine analogs for use in the treatment of HIV and other disorders.

## Supporting information

S1 Fig Serum parameters representing hepatic and renal functions after oral administration of SK14-061a in healthy rats. Rats were orally administered SK14-061a at a dose of 1 mg/kg, and observed changes in (A) aspartate aminotransferase (AST), (B) alanine aminotransferase (ALT) (C) creatinine and (D) blood urea nitrogen (BUN) at 9 hr after administration. There is no significant difference among samples. Data represents the mean ± SD. (n = 4).
(TIF)

S2 Fig Time course for the plasma concentration of SK14-061a after intravenous injection at a dose of 1 mg/kg in rats. (TIF)
