# Supplementary figures and images for: Pharmacokinetic properties of a novel inosine analog, 4′-cyano-2′-deoxyinosine, after oral administration in rats
Source: PLoS One. 2018 Jun 6;13(6):e0198636. doi: 10.1371/journal.pone.0198636 (PMC5991393; doi:10.1371/journal.pone.0198636)

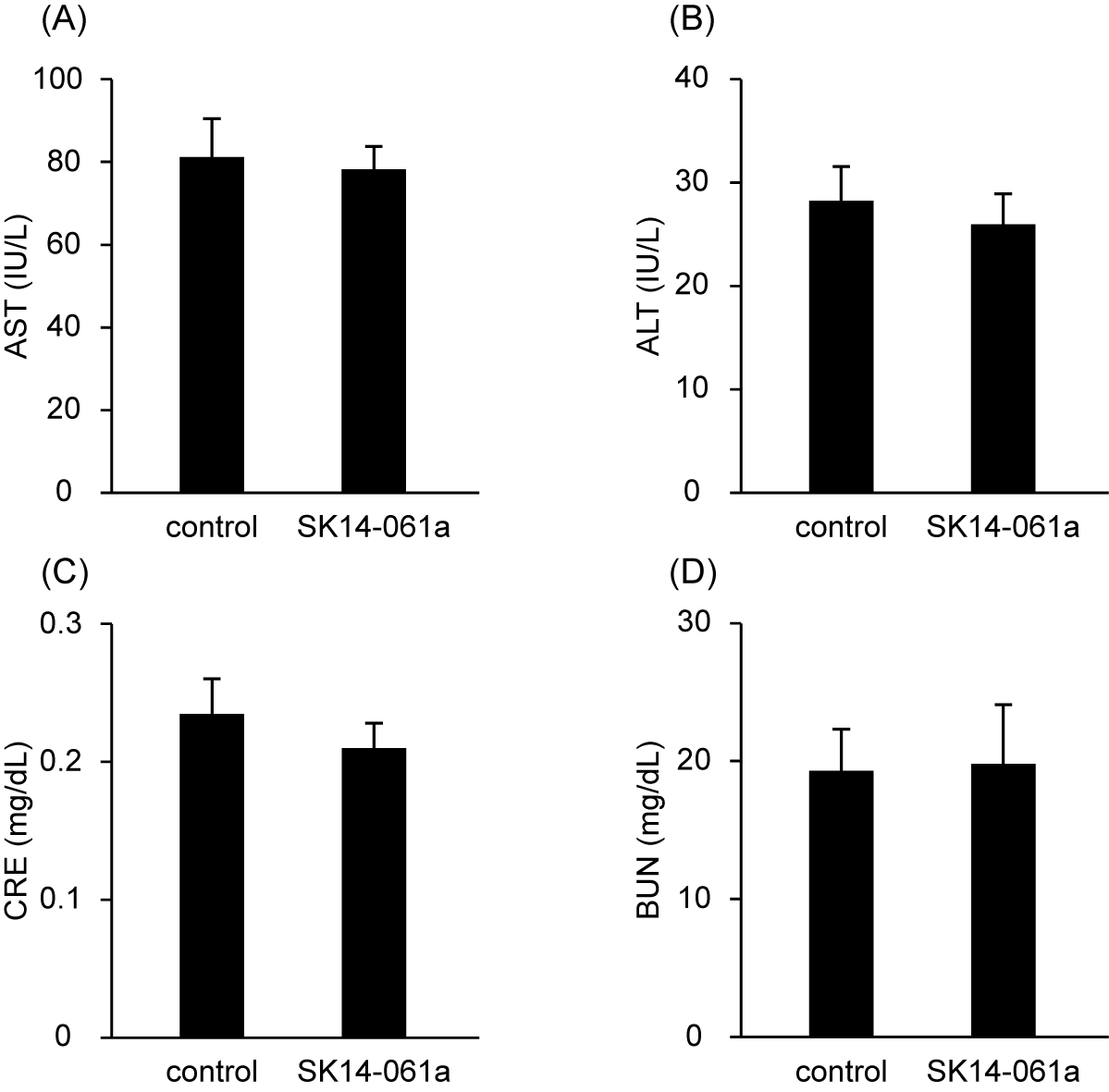

Supplement: S1 Fig — Rats were orally administered SK14-061a at a dose of 1 mg/kg, and observed changes in (A) aspartate aminotransferase (AST), (B) alanine aminotransferase (ALT) (C) creatinine and (D) blood urea nitrogen (BUN) at 9 hr after administration. There is no significant difference among samples. Data represents the mean ± SD. (n = 4). (TIF) [file pone.0198636.s001.tif]

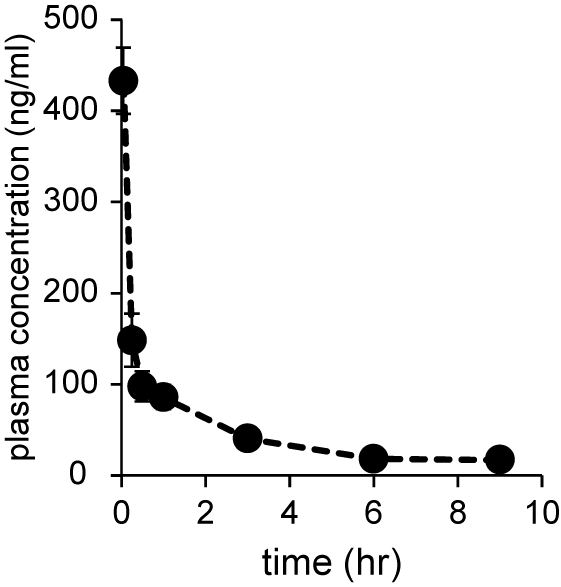

Supplement: S2 Fig — (TIF) [file pone.0198636.s002.tif]
